# Evaluation of the malaria elimination programme in Muara Enim Regency: a qualitative study from Indonesia

**DOI:** 10.1186/s12936-024-04857-7

**Published:** 2024-02-12

**Authors:** Hamzah Hasyim, Heni Marini, Misnaniarti Misnaniarti, Rostika Flora, Iche Andriyani Liberty, Ahmed Elagali, Hartoni Hartoni, Fadhilah Eka Maharani

**Affiliations:** 1 Faculty of Public Health, Universitas Sriwijaya, Indralaya, 30662 Indonesia; 2 Institute for Occupational, Social and Environmental Medicine, Faculty of Medicine at Goethe University, 60629 Frankfurt am Main, Germany; 3 Regional Technical Implementation Unit, Health Training Center (Bapelkes), Palembang, 30961 Indonesia; 4 Department of Public Health and Community Medicine, Faculty of Medicine, Universitas Sriwijaya, Palembang, 30126 Indonesia; 5 School of Biological Sciences, The University of Western Australia, Perth, 6907 Australia; 6 Minderoo Foundation, Perth, 6907 Australia; 7 Biology Department, Faculty of Mathematics and Natural Sciences, Universitas Sriwijaya, Indralaya, 30662 Indonesia

**Keywords:** Malaria, Indonesia, Healthcare, Elimination programme, Equatorial climate, Anopheles mosquitoes, Qualitative study, Challenges, Achievements, Public health

## Abstract

**Background:**

Malaria remains an enduring public health concern in Indonesia, exacerbated by its equatorial climate that fosters the proliferation of *Anopheles* mosquitoes. This study seeks to assess the performance of the malaria elimination programme comprehensively.

**Methods:**

Between May and August 2022, a qualitative study was conducted in Muara Enim Regency, South Sumatra Province, involving 22 healthcare professionals from diverse backgrounds. These informants were strategically chosen for their pivotal roles in providing profound insights into various facets of the malaria elimination programme. This encompasses inputs such as human resources, budgetary allocation, and infrastructural support; processes like case identification and management, capacity enhancement, epidemiological surveillance, prevention measures, outbreak control, and enhanced communication and educational initiatives; and, notably, the programme’s outcomes. Data were collected through 3-h Focus Group Discussions (FGDs) divided into two groups, each with 12 participants: healthcare professionals and programme managers. Additionally, in-depth interviews (IDIs) were conducted with ten informants. Employing the Input-Process-Output (IPO) model, this study meticulously analysed the healthcare system dynamics and the interventions’ efficacy.

**Results:**

The study unveiled many challenges during the input phase, including the absence of entomologists and a shortage of diagnostic tools. Despite these obstacles, it documented remarkable accomplishments in the output domain, marked by significant advancements in the distribution of mosquito nets and the successful implementation of the Early Warning System (EWS). Despite the adversities, the programme has made substantial strides towards malaria elimination.

**Conclusions:**

Urgent action is imperative to bolster the effectiveness of the malaria elimination programme. Key measures encompass augmenting the entomologist workforce, optimizing resource allocation, and ensuring stringent adherence to regional regulations. Addressing these concerns will enhance programme efficacy, yielding enduring public health benefits. This research substantially contributes to Indonesia’s ongoing malaria elimination endeavours, furnishing actionable insights for programme enhancement. Consequently, this research holds significant importance for the malaria elimination drive.

## Background

 The United Nations Sustainable Development Goal (SDG) has set a global target to eradicate malaria by 2030 [1]. However, despite the collective global efforts, malaria remains a formidable public health challenge in Indonesia, including the South Sumatra region [2–8]. Indonesia’s equatorial location facilitates the presence of malaria-receptive *Anopheles* mosquitoes, contributing to its susceptibility to malaria. A vast and dispersed population residing across over 5000 islands, significant internal migration, socio-economic disparities, and decentralized governance exacerbate this vulnerability. These multifaceted challenges demand immediate attention when combating the disease within the Indonesian context. Such efforts align with the global commitment established by the 60th World Health Assembly in 2007 and the national initiative for a malaria-free Indonesia by 2030. Actively participating in this initiative is vital for effectively mitigating the impact of malaria, considering the unique interplay of geographical, demographic, and socio-economic factors contributing to the disease’s prevalence in the country [9, 10].

The World Malaria Report’s 2023 findings in 2022 showed approximately 249 million reported malaria cases globally in 85 countries and regions endemic to malaria, indicating a significant increase of 5 million cases compared to the previous year [11]. These statistics underscore the urgency of addressing the challenges mentioned earlier. Furthermore, the World Health Organization (WHO) has outlined stringent criteria for malaria elimination certification, including an annual parasite incidence (API) of less than one per 1000 individuals, a positivity rate (PR) below 5%, and no indigenous cases for three consecutive years [12]. Achieving malaria elimination certification necessitates interrupting local transmission for all human malaria parasites. Indonesia’s government has targeted eliminating malaria by 2030, aligning with a broader regional strategy. However, Muara Enim Regency, located in South Sumatra, Indonesia, remains faced with malaria transmission. The region’s geography and climate are vital factors perpetuating the disease, presenting significant challenges to malaria control and elimination initiatives [13]. In this context, evaluating the malaria elimination initiative’s effectiveness is crucial for assessing its impact on prevalence and aligning with predetermined health objectives [14]. This study comprehensively examines the malaria elimination programme, encompassing critical aspects such as human resources, budget allocation, infrastructure development, case detection and management, capacity enhancement, epidemiological surveillance, preventive measures, outbreak control strategies, and extensive communication and educational initiatives.

Furthermore, it meticulously evaluates the programme’s outcomes, including the results of malaria elimination efforts. The research aims to deliver valuable insights and tailored recommendations for policymakers and healthcare professionals in the region. The initiative demands strategic planning, reasonable resource allocation, and the execution of targeted activities. Addressing the programme’s unique context and confronting its inherent challenges underscores the need to emphasise the successes achieved and the obstacles faced, which are pivotal for a comprehensive evaluation. In essence, this study plays a crucial role in bridging existing research gaps by meticulously assessing the effectiveness of the malaria elimination initiative, thus significantly contributing to refining and optimizing strategies employed to pursue malaria elimination. It is instrumental in addressing the pressing issue of malaria elimination in Indonesia, particularly in the Muara Enim Regency. By meticulously evaluating the effectiveness of the malaria elimination initiative, highlighting challenges, and proposing practical solutions, this research substantially contributes to the broader goal of eradicating malaria globally by 2030. It underscores the importance of tailored approaches in the fight against this disease.

## Methods

The researcher used qualitative data analysis techniques to scrutinize survey data, observational results, and information from various sources. Observations were conducted at the seven PHCs to verify the accuracy of FGDs and in-depth interview results with informants from the PHCs. The research was deductive in analysing the results of each IPO (input, process, output). At the same time, inductive analysis was based on the assessed assessment, allowing conclusions to be drawn regarding Muara Enim’s readiness to face malaria elimination. The study employed the IPO model to assess the health system systematically, providing a robust framework for evaluating specific health programmes [15]. Adopting the IPO model presents evaluators with a unified and structured approach, enhancing the precision and clarity of assessments. This framework ensures a comprehensive understanding of programme dynamics, facilitating a nuanced analysis of tailored health interventions’ overall effectiveness and impact. The IPO model for health interventions, consisting of three key stages, commences with establishing foundational resources and factors, including entomologists, diagnostic tools, and trained staff (input). The subsequent stage involves operationalizing health strategies, encompassing activities like distributing bed nets, managing the environment, and conducting epidemiological investigations (process). Noteworthy outcomes, identified as “Output,” are achieved through actions such as instituting early alert systems and progressing in eliminating mosquito breeding sites. Utilizing the IPO model provides a clear depiction of how resources and factors (input) contribute to activities and procedures (process), resulting in specific outcomes (output). This systematic approach enhances the transparency of the research process, offering a comprehensive overview of health intervention dynamics in the Muara Enim Regency.

### Study design and setting

This qualitative study involved 22 informants purposefully selected from seven public health centres (PHCs), the Muara Enim Regency Health Office, the Regional Hospital, and the Provincial Health Office in South Sumatra. Data collection was thorough and included perspectives from the informants. They employed focus group discussions (FGDs), in-depth interviews, and observation. The study encompassed a diverse group of participants across the mentioned health institutions, intentionally chosen for their insights. The study aimed to gather comprehensive insights through active engagement with informants representing various facets of the healthcare system. The variables under scrutiny encompassed input factors like human resources, budget, and infrastructure, while the process aspect involved discovering and managing sufferers, preventing and managing risk factors, epidemiological surveillance and outbreak management, improving communication, information, and education (IEC), and enhancing human resources. The study also examined output, focusing on obstacles to achieving elimination and the percentage achievement of twelve indicators in the Muara Enim Regency.

### Study site

**Fig. 1:**
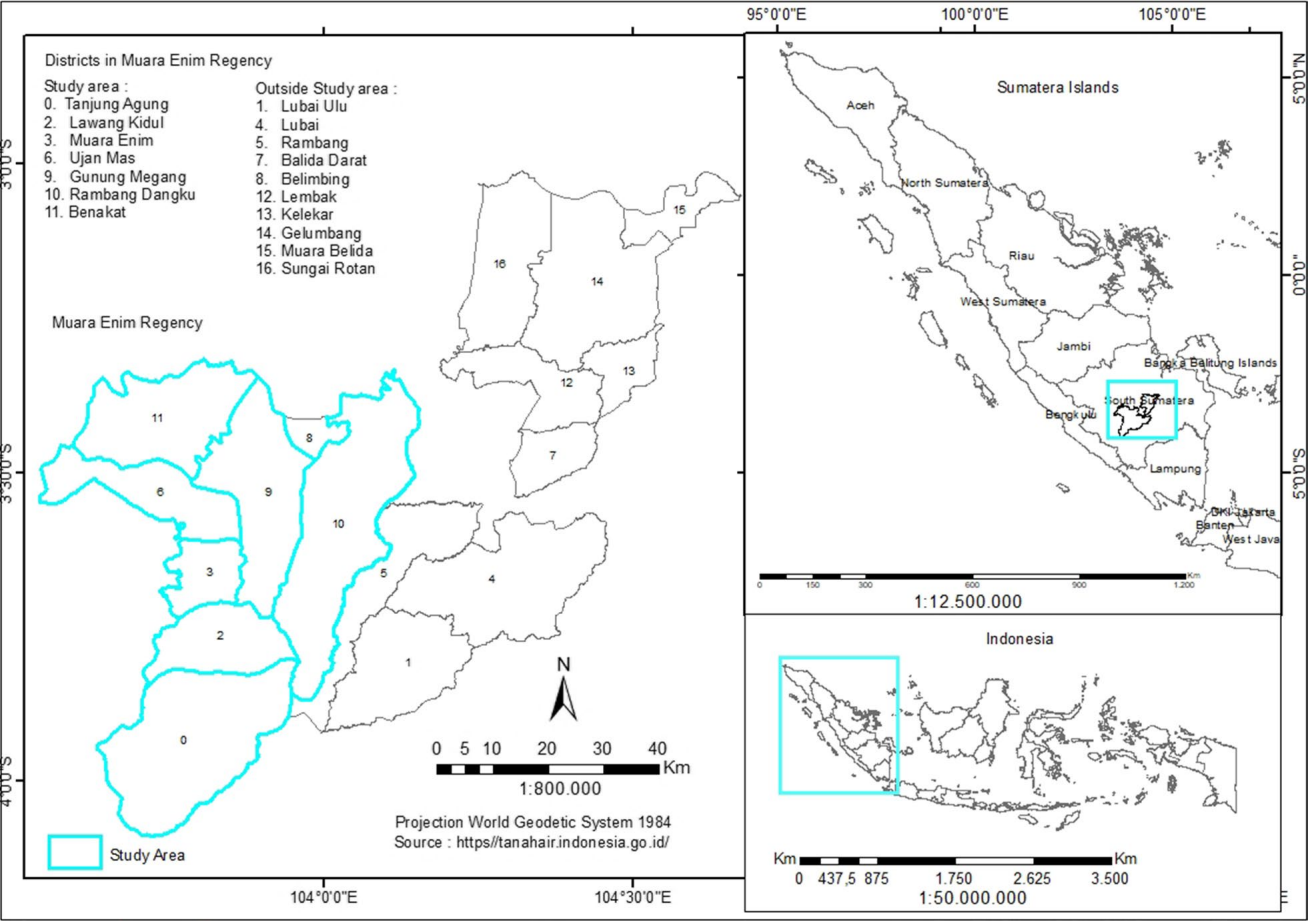
The location study

Purposive sampling determined the study locations: Muara Enim Regency Health Office, HM Rabain Hospital, and seven PHCs. The research location, Muara Enim Regency, can be seen in Fig. 1.

### Data collection

The study, conducted from May to August 2022, employed a qualitative approach for data analysis and utilized the IPO model as a framework to evaluate the health system. Data processing took place between August and October 2022. The FGD sessions were divided into two distinct groups. These FGDs were conducted by qualitative research teams from outside Muara Enim Regency. They involved twelve health workers, seven PHCs malaria programme managers, and five laboratory employees from health centres.

The primary objective of these FGDs was to assess the process of gathering and utilizing malaria-related data within PHCs and to probe the knowledge of malaria programme managers regarding the status of malaria programmes in the community. These FGDs were conducted in both Indonesian and the local Palembang languages, transcribed verbatim, translated into Indonesian when necessary, and subsequently translated into English for publication.

In addition to the FGDs, the study involved 22 key informants, including prominent figures such as the Director of Health Services, the Head of Medical Services at RSUD Rabain Muara Enim, the Head of Disease Control and Prevention (P2P), Programme Managers at the Muara Enim Regency Health Office, and Programme Managers at the South Sumatra Provincial Health Office. This group also included heads of PHCs, Malaria Programme Managers at Puskesmas, and laboratory staff at both Puskesmas and hospitals. These participants actively contributed valuable insights within their respective domains of expertise.

Furthermore, 10 informants participated in in-depth interviews, reflecting the diversity of healthcare professionals. District health office representatives, including the Head of Muara Enim Regency Health Office and the Head of the P2P Division, provided district-level perspectives and insights into programme management. The hospital and medical services sector was represented by the Head of Medical Services and a Laboratory Analyst from the Regional General Hospital Rabain, offering insights into hospital and laboratory aspects.

The strategic selection of informants was undertaken due to their crucial roles in providing profound insights into various facets of the malaria elimination programme. This encompassed aspects such as human resources, budgeting, and infrastructure, as well as processes including case detection and management, human resource development, epidemiological surveillance, prevention, outbreak response, and the enhancement of communication and education efforts. Furthermore, it extended to the programme’s outputs, precisely the outcomes of the malaria elimination programme. Integrating the findings with existing literature would strengthen this section.

### Data analysis

The recent article effectively employs both deductive and inductive reasoning to evaluate the malaria elimination programme in the Muara Enim Regency. Deductive reasoning is applied through the analysis of the IPO model as a structured framework, leading to specific conclusions about the dynamics of the health system. Simultaneously, inductive reasoning is utilized through a qualitative approach involving data collection via FGDs, in-depth interviews, and observation. This method enables the researchers to gather specific information from various sources, aiming to derive general conclusions about the effectiveness of the malaria elimination programme in the Muara Enim Regency. The study combines both deductive and inductive approaches, providing a comprehensive evaluation of the health intervention. The coding procedure is executed, and the acquired process is iterated by identifying the central concept of the comprehended coding. Initially, categories are meticulously established to minimize potential overlap, followed by the researcher’s comprehensive assessment of all relevant codes. The last phase involves categorizing all codes with the same semantic content and assigning names that accurately reflect the meaning encapsulated inside each code. Analysis refers to systematically examining and evaluating data or information to gain a deeper understanding, identify patterns, and draw meaningful conclusions. The researcher engages in an iterative examination of the narrative data to ascertain significance and comprehension. In addition to transcribing the tape, significant data collection is supplemented with observational notes. The process involves documenting concepts and capturing ideas as a foundation for categorization, data reduction, and subsequent data mapping and interpretation. Subsequently, a matrix is constructed to identify relationships between each interpretation and discern the associations between different variables (Table 1).

**Table 1:** Seven PHCs within the same hierarchical structure were chosen based on the working area of the API high

Public Health Centre	Annual paracite incidence (API)	Malaria Cases
PHC Ujan mas	0.12 (2019)	3 (2019)
PHC Benakat	0.75 (2018), 0.19 (2019), 0.1 (2020)	8 (2018), 2 (2019), 1 (2020)
PHC Gunung Megang	0.15 (2018)	4 (2018)
PHC Muara Enim	0.1 (2019)	7 (2019)
PHC Tebat Agung	0.11 (2022)	4 (2022)
PHC Tanjung Agung	0.7 (2018), 0.49 (2019), 0.05 (2020)	29 (2018), 20 (2019), 2 (2020)
PHC Tanjung Enim	0.83 (2018), 0.31 (2019), 0.13 (2020), 0.09 (2022)	56 (2018), 21 (2019), 9 (2020), 6 (2022)

The research team selected malaria cases for study participation from 2018, 2019, 2020, and 2022

The annual parasite incidence (API) and malaria cases at the Primary Health Centres (PHCs) in Muara Enim Regency exhibit significant variation. PHC Ujan Mas reported an API rate of 0.12 in 2019, with three recorded malaria cases. Conversely, PHC Benakat recorded fluctuating API rates, namely 0.75 (2018), 0.19 (2019), and 0.1 (2020), with corresponding case numbers of eight (2018), two (2019), and one (2020). PHC Gunung Megang reported an API rate of 0.15 in 2018, with four documented malaria cases. Furthermore, PHC Muara Enim recorded an API rate of 0.1 in 2019, with seven reported malaria cases. PHC Tebat Agung reported an API rate of 0.11 in 2022 with four malaria cases documented. At PHC Tanjung Agung, there was fluctuation in the API rate, specifically 0.7 (2018), 0.49 (2019), and 0.05 (2020), with corresponding case numbers of 29 (2018), 20 (2019), and 2 (2020). Meanwhile, PHC Tanjung Enim documented fluctuating API rates as well, namely 0.83 (2018), 0.31 (2019), 0.13 (2020), and 0.09 (2022), with corresponding case numbers of 56 (2018), 21 (2019), 9 (2020), and 6 (2022)

This data provides insight into the complexity and distribution of malaria cases across various PHCs, serving as a foundation for further intervention planning in malaria control efforts in this region

In the study area, the identified PHCs included Ujan Mas, Benakat, Gunung Megang Tebat Agung, Muara Enim, Tanjung Agung, and Tanjung Enim PHCs. The selection of informants in this study adhered to the principles of suitability and adequacy. The researcher purposefully chose informants by assessing their capacity to provide the desired information or data. This process involved evaluating their appropriateness for the study. The researchers selected key informants based on the theories and data possessed by these individuals. Consequently, the informants for this study comprised the Head of the District Health Office of Muara Enim, the head of the service division at HM Rabain Hospital Muara Enim, the head of disease control and prevention (P2P) at the Muara Enim Regency Health Office, the Program Manager of the District Health Office of Muara Enim, two heads of PHCs, seven program managers for malaria in PHCs, seven PHC Laboratory Officers, one hospital laboratory officer, and a key informant, namely the Program Manager of the South Sumatra Provincial Health Office

### Ethical considerations

The research protocol was approved by the Health Research Ethics Committee of the Faculty of Public Health, Universitas Sriwijaya, with Ethical Approval No: 251/UN9.FKM/TU.KKE/2022 participation in this study was highly voluntary in the field. All analyses were performed using participant identification codes to ensure maximum confidentiality.

## Results

In this study, informants were selected based on the appropriateness principle, signifying their selection for their knowledge relevant to the research topic. The non-probability purposive sampling technique was applied. The informants in this research served as primary data sources acquired through in-depth interviews and FGDs. They included the Head of the Muara Enim Regency Health Office, the Head of Medical Services at Rabain Muara Enim Regional General Hospital, the Head of the Division for Disease Control and Prevention (P2P) at the Muara Enim Regency Health Office, Malaria Programme Managers from both the Muara Enim Regency Health Office and the South Sumatra Provincial Health Office, Heads of PHCs, PHCs Laboratory Analysts, and Hospital Laboratory Analysts. The characteristics of the informants are detailed in Table 2.

**Table 2:** Characteristics of research informants

Informant details	Type of discussion	Code	Gender	Age (years)	Education	Position
FGD 1 (Malaria Program Managers - PHC)	Malaria Program Manager PHC	A1	P	47	D3	Malaria Program Manager, PHCs Ujan Mas
		A2	P	42	S1	Malaria Program Manager, PHCs Benakat
		A3	P	53	D3	Malaria Program Manager, PHCs Gunung Megang
		A4	P	29	D3	Malaria Program Manager, PHCs Tebat Agung
		A5	P	45	D3	Malaria Program Manager, PHCs Muara Enim
		A6	P	46	D3	Malaria Program Manager, PHCs Tanjung Agung
		A7	P	34	D3	Malaria Program Manager, PHCs Tanjung Enim
FGD 2 (PHC Laboratory Technicians)	PHC Laboratory Analysts	B1	L	33	D3	Laboratory Analyst, PHCs Ujan Mas
		B2	P	54	D3	Laboratory Analyst, PHCs Benakat
		B3	P	30	D3	Laboratory Analyst, PHCs Gunung Megang
		B4	P	31	D3	Laboratory Analyst, PHCs Tanjung Enim
		B5	P	24	D3	Laboratory Analyst, PHCs Muara Enim
In-depth interviews	–	C1	L	53	S2	Head of Muara Enim Regency Health Office
		C2	L	52	S2	Head of Medical Services, Regional General Hospital Rabain
		C3	P	54	D3	Head of P2P Division, Muara Enim Health Office
		C4	L	40	S1	Malaria Program Manager, Muara Enim Health Office
		C5	L	40	S2	Malaria Program Manager, South Sumatra Provincial Health Office
		C6	P	58	S1	Head of PHCs Benakat
		C7	L	58	S1	Head of PHCs Tanjung Enim
		C8	P	33	D3	Laboratory Analyst, PHCs Tanjung Agung
		C9	P	35	D3	Laboratory Analyst, PHCs Tebat Agung
		C10	P	41	D3	Laboratory Analyst, regional general hospital Rabain

The table above outlines the presence of 22 informants in this study, encompassing Heads, Department Heads, and personnel from the Muara Enim Regency Health Office. Additionally, it includes health professionals from 7 Primary Health Centres (PHCs) in Muara Enim and Department Heads with staff from the regional general hospital Rabain Muara Enim. This inclusive table amalgamates the characteristics of the study’s respondents, offering insights into their roles, backgrounds, and contributions. Through diverse interview methods, these key figures shared valuable information about the essential components of the malaria elimination program in Muara Enim. The structured approach, coupled with a diverse range of respondents, ensures a comprehensive understanding of the challenges and potential solutions within the health system

### Inputs

The qualitative assessment highlights the critical role of human resources in the malaria programme. Malaria programme management officers strategically operate in PHCs and Regency health offices to oversee programme implementation. However, a significant gap exists in deploying entomologist personnel at PHCs and regency hospitals. Some PHCs laboratory officers lack training in malaria microscopy, primarily due to staff transfers, and the presence of newly hired personnel compounds the issue. Among the 22 PHCs, only two have exclusive malaria cadres. Budget-wise, the malaria programme in Muara Enim Regency has depended on government funds and private sector contributions, such as global funds and assistance from private companies, since 2018. However, in 2022, regional governments face minimal funding due to the absence of reported malaria cases in the previous year. Infrastructure is also crucial, with the absence of a malaria screening tool like a Rapid Diagnostic Test (RDT) posing a challenge at health centres. This challenge is particularly noteworthy as no cases have been reported in recent years, making it a lower priority for PHCs, as highlighted by the head of the PHCs during an in-depth interview.

Observations at various health centres revealed inaccessibility to RDTs at PHCs due to insufficient supply for the entire village area. To address this, the PHCs decided to procure RDTs using funds from the PHCs (regional public service agency), as suggested by the head of the regency health office. Among the seven PHCs observed in the field, it was found that six possessed unsuitable or expired Giemsa reagents. Consequently, microscopic examinations have not been conducted over the past two months at these PHCs despite having functional microscopes. An informant from the PHCs laboratory disclosed that the laboratory does not meet the required standards for malaria examination. Specifically, the laboratory lacks running water, some areas lack proper equipment for microscopy, and the malaria examination site is still integrated with other examination facilities.

### Process

The identification and management of individuals rely on timely patient detection, accurate diagnosis, effective management, and the stabilization of microscopic characteristics. Insights from FGDs and document analysis reveal a dual approach to case discovery, incorporating both active and passive methods. Active Mass Blood Survey (MBS) and Active Case Detection (ACD) methods are deployed for proactive disease surveillance, complemented by recommended strategies such as epidemiological investigations, follow-up treatment contact surveys, and migration surveillance. Emphasis is placed on passive case detection (PCD) methods, selective malaria screening for pregnant women, and examination services for ill toddlers through the Integrated Management of Childhood Illness approach. The diagnosis involves RDT and microscopic examination. Notably, RDT utilization is restricted to field settings and is not widely adopted at PHCs, where laboratory facilities are available for malaria testing, as highlighted by informants from the Regency Health Service.

At PHCs, the treatment of Malaria-positive patients adheres to national standards based on the type of malaria parasite identified. The malaria programme manager conducts follow-up treatment tailored to the specific species of *Plasmodium* found in the patient.

Implementing measures to control risk factors is crucial in breaking the malaria transmission cycle, as highlighted in FGDs. Various strategies have been employed, encompassing preventive measures against mosquito bites, managing malaria vectors, and monitoring vector populations. Actively implemented initiatives such as environmental clean-up, collaborative community events in sub-regions, larvicide powder distribution, the introduction of mosquito larvae-eating fish, indoor residual spraying (IRS) in the Tanjung Agung region, and the distribution of insecticide-treated mosquito nets (LLINs) to diagnosed malaria patients and pregnant women showcase the concerted efforts. In 2019, the Benakat Health Centre area actively participated in vector surveillance by periodically monitoring larvae at five locations with involvement from village officials, specifically targeting areas where mosquito breeding was observed. The surveillance included mosquito capture activities, and the team analysed and mapped the collected data using the Malaria Surveillance Information System (SISMAL) programme to identify areas needing targeted vector control measures. PHCs face challenges as they lack access to receptive maps. In an in-depth interview, the chief of the Regency Health office identified community behaviour as a significant challenge, persisting as a continuous concern in the pursuit of malaria elimination.

As per the informant’s account, there is a prevailing belief among the people that mosquito bites are considered a common occurrence. Additionally, the informant mentioned that nearly every household in the community possesses mosquito nets. However, these mosquito nets are often repurposed for use as fishing nets. As a result, mosquito larvae are in the area due to the community’s practice of collecting rainwater for their daily needs.

Epidemiological surveillance and outbreak control involve a series of interconnected steps, including reporting positive cases, conducting thorough epidemiological investigations, creating area maps, implementing necessary outbreak control measures, and employing targeted case-finding methods in specific priority areas. The PE 1-2-5 method requires reporting a positive malaria case to the regency/city health office within 24 h to streamline epidemiological investigations. However, challenges arise in executing this method, with the initial reporting meeting the 24-h timeframe but the epidemiological investigation occurring on the third day. Public Health Education, a collaborative effort between the Regency Health Office and the Public Health Centre, involved a contact survey focusing on 25 individuals with similar risk factors as malaria cases. Further investigations and classifications were conducted to narrow the scope of inquiry, and the malaria programme manager implemented preventive measures, such as distributing insecticide-treated nets. Area mapping, facilitated by the SISMAL application, identified geographical locations of malaria cases reported to the Regency Health Office. The management of outbreaks is facilitated by submitting weekly reports from the PHCs to the Regency Health Office using the SISMAL. The encountered limitations at the Regency Health Office are outlined.

*“When dealing with issues related to Epidemiological Investigation (EI), it is essential to note that hospital staff sometimes fail to report cases promptly. It may come as a surprise that reports from the Hospital Information Management (HIM) department are only received the following month, leaving the team uninformed until then. Currently, we are awaiting an update on the report.*

*However, Swift action is necessary if the report originates from the Primary Health Centre (PHC). Upon receiving a report from the PHC today, promptly implement the established 1-2-5 method and proceed accordingly. Conversely, if a report from the hospital must be awaited by next month, schedule a visit to verify patients’ adherence to prescribed medication and assess the completeness of the medication provided.*

*Examinations generally commence following the 1-2-5 method. At a minimum, interviews are conducted to ascertain whether family members or neighbors have displayed fever symptoms. If anyone exhibits fever symptoms, a medical examination is performed. The approach varies when there is prior knowledge of a specific case; in such instances, an immediate examination is carried out, even if the patient is not currently experiencing a fever. The primary focus remains on the 25 individuals close to the patient, including friends and close acquaintances”.*

The results of the in-depth interview stated that the reporting of positive cases at the hospital was carried out in the following month, so no one carried out an epidemiological investigation. But only follow up with sufferers in the form of interviews with sufferers.

The enhancement of IEC is cross programmed, with educational campaigns complementing screening sessions focused on malaria prevention. Collaborative efforts involve various sectors, including mining corporations, to disseminate counselling materials emphasizing rain tank drum management, proper closure, cleaning practices, and keeping cattle away from residential areas. Some PHCs establish cross-border partnerships, while mining corporations in the Tanjung Enim region offer counselling services. Advocacy directed at mining firms aims to secure financial resources for malaria control. Public-private partnerships, supported by memorandums of understanding, involve health facilities in Muara Enim, collaborating with governmental entities like the Ministry of Health and the Provincial Health Service for funding. Partnerships extend to mosquito control, quality assurance checks, cross-check examinations, and cross-notification processes with various institutions. Inadequate educational resources on malaria persist in specific PHCs, as seen in the absence of local educational materials. The augmentation of human resources involves revitalizing services for healthcare professionals, addressing gaps in training for malaria programme managers and laboratory personnel, and reorganized under the Regency Health Office to manage the malaria programme.

It can be concluded that not all officers have received training on malaria.

### Output

Numerous challenges impede the goal of malaria elimination in the Muara Enim Regency. These include the presence of rainwater reservoirs, such as large drums in front of residents’ houses, clogged gutters fostering stagnant water accumulation, unused fishponds contributing to additional stagnant water sources, and swamps providing ideal breeding grounds for mosquitoes. Inadequate management of cattle farms further exacerbates the malaria persistence issue. Challenges also arise from the lack of on-site laboratory personnel and delays in obtaining examination results from the RDT. Additionally, community engagement for vector control proves challenging due to the infrequent gatherings of individuals engaged in gardening activities, returning to the village only once a week. A potential strategy for achieving malaria elimination involves providing training and support to malaria programme managers to enhance their knowledge and skills. In epidemiological investigations, fostering collaboration with various sectors, including village or local area officials, is imperative to address individuals’ apprehensions during participation in contact surveys. The overall achievement rate of the 12 indicators in Muara Enim Regency towards malaria elimination stands at 86.56%. An authority set the standard assessment scores for malaria elimination tools in Muara Enim Regency in 2022 at 20 for districts, 19.25% for PHCs, and 8.75% for hospitals. The results obtained in the same year revealed assessment scores of 17.5, equivalent to 87.5%, 16.32, representing 84.77%, and 7.65, amounting to 87.42% for districts, health PHCs and hospitals, respectively. Consequently, the overall average assessment score for malaria elimination tools, reflecting the comprehensive achievement level across twelve indicators, reached a commendable figure of 86.56%. This substantial achievement underscores the district’s commitment to combat malaria effectively.

## Discussion

### Main findings

This study provides crucial insights into the malaria elimination programme in Muara Enim Regency, South Sumatra Province, Indonesia. It addresses critical facets of the programme, from human resources to infrastructure, case management to preventive measures, and highlights achievements and challenges.

These findings align with existing research, emphasizing the importance of robust human resources in malaria control and elimination. The absence of entomologists and limited diagnostic tools mirror challenges faced in other malaria-endemic regions. Flexible funding practices at sub-national levels resonate with the need for financial sustainability highlighted in previous studies. Infrastructure deficiencies and the need for comprehensive approaches are consistent themes in malaria research. The confirmation of fulfilling prerequisites for malaria elimination aligns with global goals set by the WHO.

Human resources for the malaria programme in Muara Enim Regency are generally adequate. Still, there is a notable absence of entomology staff at the PHCs and entomology assistant staff at the Regency, impacting the functional aspect of entomologists. Only 9% of PHCs have laboratory analysts trained in malaria microscopy, emphasizing the need for enhanced expertise. Specialized malaria cadres are available at specific health centres. Another study stresses the importance of bolstering appropriate human resources at all levels to sustain progress and expedite malaria control and elimination [16]. Muara Enim Regency faces budgetary constraints for malaria in 2022 due to minimal regional revenues. Inadequate financing, necessary behavioural changes in the community, and the absence of sustainable digital tools contribute to the slow progress in malaria elimination. The study recommends that donors adopt practices facilitating flexible funding at sub-national levels, integrating case management, vector control, surveillance, and evaluation to enhance the effectiveness of malaria control and elimination efforts [17, 18]. . However, not all PHCs meet laboratory room standards in Muara Enim Regency, and some lack reagents for microscopic malaria examinations, underlining the need for improved infrastructure. A comprehensive malaria elimination programme necessitates strategic planning, personnel expertise, infrastructure development, collaborations, and consistent financial support, with active participation from government officials to overcome technological, financial, and political challenges [19]. In the Muara Enim regency, malaria research utilises RDT and microscopic examination techniques, confirming positive cases through microscopy and supporting the efficacy of this diagnostic approach [20]. Active and passive methods for malaria case identification involve the MBS system, contact surveys, and migration surveillance by three specific health facilities [21]. Treatment aligns with government protocols, delivering artemisinin-based combination therapy and primaquine supplementation, proven effective for all malaria types [22]. Prevention strategies include insecticide-treated net distribution, allocating nets to positive individuals and pregnant women in the first trimester, and addressing the risk of non-utilization at night [23].

Larvicide implementation aims for collaborative efforts, utilising larvae-consuming fish species for reclamation in ex-mining sites [24]. Epidemiological surveillance involves various activities, but challenges arise from unreported positive cases, hindering timely information dissemination to the PHC’s malaria programme manager [25]. Systematic scaling-up is crucial in malaria-endemic areas, involving research surveillance, 1-2-5 epidemiological investigations, case management network enhancement, and vector population reduction. The health information system relies on specific applications like SISMAL, with practical limitations due to internet constraints. Communication and education efforts in Muara Enim Regency lack a regional regulation for malaria elimination, differing from initiatives in West Sumatra Province. The Health Office collaborates with mining firms and four health clinics, emphasising the necessity of strengthening cross-sector collaboration [26]. Four of seven health centres possess informational booklets about malaria counselling, while none own local extension media. The study highlights a lack of malaria microscopy training for laboratory analysts since 2019, necessitating updated human resources, and managers overseeing the malaria programme at PHCs lack training engagement, potentially attributed to a new management approach [27, 28]. Upon analysing observations, document reviews, and consultations with the malaria programme manager at the Provincial Health Office, it is confirmed that Muara Enim Regency fulfils the three prerequisites for malaria elimination, as indicated by the API and PR values. The instrument used in the 2022 malaria elimination evaluation yielded a result exceeding 86.56%. This tool meets the criteria for pre-assessment in the context of malaria elimination. Muara Enim Regency successfully meets the requirements for malaria elimination, evidenced by achieving an API value of less than one, a slide positivity rate of less than five, and the absence of indigenous cases from 2020 to 2022. Malaria elimination is defined as the purposeful reduction to zero incidence of native cases of a specified malaria parasite species within a designated geographical area, demanding sustained measures to forestall the re-establishment of transmission. Certification of malaria elimination in a country necessitates the cessation of local transmission for the four principal human malaria parasites [29]. Moreover, the WHO awards malaria elimination certification to countries that effectively demonstrate the interruption of local transmission for all human malaria parasites over a minimum period of three consecutive years [30].

### Limitations of the study

In light of the identified limitations, it is imperative to acknowledge the qualitative nature of this research, which may constrain the generalizability of its findings. The study’s reliance on healthcare professionals and programme managers as primary informants might introduce a potential source of bias in the collected data. Furthermore, the study’s limited geographic scope, focused solely on a specific region in Indonesia, raises questions about the universal applicability of its conclusions. To effectively address these limitations, future research endeavours should consider diversifying data sources and methodologies, embracing a more inclusive approach incorporating community perspectives, and expanding the study’s geographic scope to provide a broader understanding of malaria elimination efforts.

## Conclusion

In conclusion, evaluating the Malaria Elimination Programme in Muara Enim Regency reveals commendable progress and critical challenges. Using the Input-Process-Output (IPO) model, this comprehensive qualitative study provides valuable insights into programme implementation. The study highlights several strengths and weaknesses. A remarkable success rate of 86.56% across twelve malaria indicators, encompassing districts, hospitals, and research health institutes, is a notable achievement. Additionally, consistently low values for the API, the slide positivity rate, and the absence of indigenous malaria cases signify significant progress towards stringent criteria for malaria elimination. However, challenges include a shortage of entomologists, limited resources, regional non-compliance with epidemiological investigations, insufficiently trained management personnel, and laboratory facilities and materials constraints. These challenges need practical solutions. To address these issues, recruiting more entomologists, allocating adequate resources, and providing targeted training. Regional compliance with epidemiological investigations and regulations is crucial for robust malaria control.

Furthermore, the programme’s success in Muara Enim Regency has broader implications for malaria prevention efforts, suggesting the feasibility of malaria elimination in other regions through effective strategies. The study’s findings offer valuable guidance to policymakers for resource allocation, promoting best practices, and formulating policy recommendations at both regional and national levels. While Muara Enim Regency has made significant strides in malaria prevention, sustained efforts are vital to address existing challenges and enhance the programme’s effectiveness.

A comprehensive examination of the long-term sustainability of the programme’s success will offer valuable insights for future initiatives. In summary, this research establishes a solid foundation for maintaining malaria control and elimination efforts, advancing public health regionally and nationally, and serving as a critical reference for enhancing malaria control and elimination programmes in various regions. These findings emphasize the importance of ongoing commitment and adapting proven successful strategies for a broader impact on public health.

## Data Availability

The qualitative data supporting this study’s findings are not publicly available, as this would compromise participant privacy. Participants did not consent to have their interview transcripts made publicly available. However, data may be available from the corresponding author upon reasonable request.

## Abbreviations

ACD: Active case detection
API: The Annual parasite incidence
BTKLPP: The Environmental Health and Disease Control Engineering
CIE: Communication, information, and education
IEC: Improving communication, information, and education
IRS: Indoor residual spraying
LLINs: Long-lasting insecticidal nets
MBS: Mass blood survey
MCH: Maternal and child health
PCD: Passive case detection
PHCs: Primary Health Centres
PR: Positivity rate
RDT: Rapid diagnostic test
SDGs: The Sustainable development goals
SISMAL: Malaria surveillance information system
WHO: World Health Organization

## References

[1] Micah AE, Su Y, Bachmeier SD, Chapin A, Cogswell IE, Crosby SW (2020). Health sector spending and spending on HIV/AIDS, tuberculosis, and malaria, and development assistance for health: progress towards sustainable development goal 3. Lancet.

[2] Ahmad RA, Ferdiana A, Surendra H, Sy TR, Herbianto D, Rahayujati TB (2021). A participatory approach to address within-country cross-border malaria: the case of Menoreh hills in Java, Indonesia. Malar J.

[3] Hasyim H, Nursafingi A, Haque U, Montag D, Groneberg DA, Dhimal M (2018). Spatial modelling of malaria cases associated with environmental factors in South Sumatra, Indonesia. Malar J.

[4] Hasyim H, Dhimal M, Bauer J, Montag D, Groneberg DA, Kuch U (2018). Does livestock protect from malaria or facilitate malaria prevalence? A cross-sectional study in endemic rural areas of Indonesia. Malar J.

[5] Hasyim H, Dale P, Groneberg DA, Kuch U, Müller R (2019). Social determinants of malaria in an endemic area of Indonesia. Malar J.

[6] Hasyim H, Firdaus F, Prabawa A, Dale P, Harapan H, Groneberg DA (2020). Potential for a web-based management information system to improve malaria control: an exploratory study in the Lahat district, South Sumatra province, Indonesia. PLoS ONE.

[7] Hasyim H, Dewi W, Lestari R, Flora R, Novrikasari N, Liberty I et al. Malaria among mine workers in Tanjung Agung Muara Enim Regency, South Sumatra, Indonesia: an analysis of environmental risk factors. In: Proceedings of 3rd Sriwijaya International Conference on Environmental Issues, October 5th, 2022, Palembang, South Sumatera, Indonesia.

[8] Hasyim H, Ihram MA, Fakhriyatiningrum Misnaniarti, Idris H, Liberty IA (2023). Environmental determinants and risk behaviour in the case of indigenous malaria in Muara Enim regency, Indonesia a case-control design. PLoS ONE.

[9] WHO (2023). Overview of malaria.

[10] Lee J, Ryu JS (2019). Current status of parasite infections in Indonesia: a literature review. Korean J Parasitol.

[11] WHO (2023). World malaria report 2023.

[12] Kemenkes RI. Petunjuk teknis penilaian eliminasi malaria Kabupaten/Kota Dan Provinsi. Jakarta. Direktorat Pencegahan Dan Pengendalian Penyakit Tular Vektor Dan Zoonotik. Direktorat Jenderal Pencegahan Dan Pengendalian Penyakit, Kemenkes RI, Jakarta; 2023.

[13] Hasyim H, Dewi WC, Lestari RAF, Flora R, Novrikasari N, Liberty IA (2023). Risk factors of malaria transmission in mining workers in Muara Enim, South Sumatra, Indonesia. Sci Rep.

[14] Visa TI, Ajumobi O, Bamgboye E, Ajayi I, Nguku P (2020). Evaluation of malaria surveillance system in Kano state, Nigeria, 2013–2016. Infect Dis Poverty.

[15] Sulistyawati S, Sofiana L, Amala SK, Rokhmayanti R, Astuti FD, Nurfita D (2020). Pneumonia a neglected disease: a mixed-method study on the case-finding program in Indonesia. AIMS Public Health.

[16] Mwenesi H, Mbogo C, Casamitjana N, Castro MC, Itoe MA, Okonofua F (2022). Rethinking human resources and capacity building needs for malaria control and elimination in Africa. PLoS Glob Public Health.

[17] Mbunge E, Millham R, Sibiya N, Takavarasha S (2021). Is malaria elimination a distant dream? Reconsidering malaria elimination strategies in Zimbabwe. Public Health Pract.

[18] Gosling R, Chimumbwa J, Uusiku P, Rossi S, Ntuku H, Harvard K (2020). District-level approach for tailoring and targeting interventions: a new path for malaria control and elimination. Malar J.

[19] Cao J, Newby G, Cotter C, Hsiang MS, Larson E, Tatarsky A (2021). Achieving malaria elimination in China. Lancet Public Health.

[20] Oyegoke OO, Maharaj L, Akoniyon OP, Kwoji I, Roux AT, Adewumi TS (2022). Malaria diagnostic methods with the elimination goal in view. Parasitol Res.

[21] Azizi H, Davtalab-Esmaeili E, Farahbakhsh M, Zeinolabedini M, Mirzaei Y, Mirzapour M (2020). Malaria situation in a clear area of Iran: an approach for the better understanding of the health service providers’ readiness and challenges for malaria elimination in clear areas. Malar J.

[22] Kurup N, Rajnani N, Kendrekar P (2022). Chronology of drug development for malaria. Drug development for malaria: novel approaches for prevention and treatment.

[23] Nkunzimana E (2020). Knowledge and utilisation of intermittent preventive treatment of malaria among pregnant women in Muramvya Health district, Burundi, 2018. East Afr Health Res J.

[24] Mutero CM, Okoyo C, Girma M, Mwangangi J, Kibe L, Ng’ang’a P (2020). Evaluating the impact of larviciding with Bti and community education and mobilisation as supplementary integrated vector management interventions for malaria control in Kenya and Ethiopia. Malar J.

[25] Ridha MR, Fakhrizal D, Hidayat S, Liani E (2021). An overview of malaria elimination efforts in South Kalimantan from 2010 to 2018. Int J Public Health.

[26] Asmiani A, Windusari Y, Hasyim H (2021). Malaria vector control and the electronic malaria surveillance information system (E-SISMAL) in Bangka Barat regency Indonesia. J Kesehatan Lingkungan.

[27] Maturana CR, de Oliveira AD, Nadal S, Bilalli B, Serrat FZ, Soley ME (2022). Advances and challenges in automated malaria diagnosis using digital microscopy imaging with artificial intelligence tools: a review. Front Microbiol.

[28] George M (2020). The fragmentation and weakening of institutions of primary healthcare. Econ Political Weekly.

[29] WHO (2020). Preparing for certification of malaria elimination.

[30] WHO (2023). Malaria elimination certification process.

